# Growth Mindset Intervention's Impact on Positive Response to eHealth for Older Adults With Chronic Disease: Randomized Controlled Trial

**DOI:** 10.2196/65519

**Published:** 2025-07-03

**Authors:** Meijuan Cao, Xiaojuan Xu, Yaling Zeng, Binyu Zhao, Chunqi Xie, Hailu Wu, Jianlin Lou

**Affiliations:** 1 School of Medicine and Nursing, Huzhou University, 759 Erhuan Rd, Huzhou, 313000, China, 86 13656654679; 2 School of Nursing, Hangzhou Normal University, Hangzhou, China

**Keywords:** e-health, older adults, chronic diseases, growth mindset intervention, positive response, randomized controlled trial

## Abstract

**Background:**

Although eHealth has shown promise in managing chronic diseases, there remains a substantial digital divide among older adults. The concept of a growth mindset, based on psychological theory, offers a new direction and potential breakthrough for addressing this dilemma.

**Objective:**

This study aims to develop and explore the feasibility and efficacy of a growth mindset intervention for older adults with chronic diseases and their positive response to eHealth.

**Methods:**

A randomized controlled trial was conducted at the internal medicine departments of a hospital in Hangzhou, Zhejiang Province, China, from September 2021 to October 2022. A total of 77 older patients with chronic disease initially participated in the study. The mean age of the participants was 67.16 (SD 7.04) years, with 42.86% (33/77) being women and 57.14% (44/77) being men. The experimental group received an eHealth program intervention plus a growth mindset intervention over 12 weeks, with weekly sessions for the first 6 weeks and biweekly follow-up phone calls for the next 6 weeks. Each session lasted at least 25‐45 minutes. Data were collected using a personal information form, the Implicit Theories of Intelligence Scale-6 (ITIS-6), and a questionnaire on knowledge, willingness, confidence, and practice of smart medicine (KWCP-SM). Measurements were taken at the beginning of the study (T0), immediately after the 6 weeks of training provided to the experimental group (T1), and after the 12 weeks of training for the intervention (T2). Data were analyzed using repeated-measures analysis of variance and analysis of covariance.

**Results:**

The final sample comprised 74 participants, of which 36 were in the experimental group and 38 in the control group. After 12 weeks of intervention, the level of growth mindset was significantly higher in the intervention group (*P*<.05) and significant group × time interaction was observed (Wald=11.57; *P*<.05) between the two groups. KWCP-SM scores increased in both groups (*P*<.05), with more significant changes in the intervention group.

**Conclusions:**

This study demonstrated the effectiveness of the intervention program in improving the growth mindset level of older adults with chronic diseases and bridging the “digital divide” among them. Future studies should refine this intervention, considering the characteristics and needs of this population, to create fault-tolerant and lifelong growth environments that enhance growth mindset in older adults.

## Introduction

Chronic disease is one of the major causes of morbidity and mortality worldwide, which occurs predominantly among older adults [1]. According to the Chinese Bureau of Statistics, the population aged 60 years and older reached 260 million in 2020, while 75% were diagnosed with at least one chronic disease [2,3]. As a health-vulnerable group, older adults with chronic disease require long-term health care service and self-management to adapt to physiological changes, posing a tremendous burden on patients, caregivers, and the health care system [4-6], and this is where eHealth can contribute.

eHealth is characterized by information technology, automation, and intelligence, and enables patients, especially those with chronic disease, to monitor their health parameters automatically, receive quality clinical care remotely, and achieve health management goals through interactions with eHealth systems, medical personnel, and health care institutions [7,8]. It is regarded as an efficient approach to solve the uneven distribution of limited medical resources and reduce the burden of health care expenses [7-9]. By 2020 , more than 5000 hospitals in China provided online medical services, and over 900 internet hospitals were established, covering national prefecture-level cities [10]. Studies have shown that eHealth such as eHealth apps, monitoring devices [11], wearable devices [12], and rehabilitation systems [13], can improve the outcomes of chronic disease significantly. However, patients with chronic diseases in old age, especially those with age-related stereotypes and fixed mindsets, possess a low degree of willingness and confidence in using eHealth services. Vailati Riboni and Pagnini [14] found that negative stereotypes can trigger stereotype threat, causing older adults to experience anxiety and inferiority when encountering new technologies, thereby impairing their operational abilities. They frequently struggle to comprehend overwhelming and boring information offered across eHealth services [15,16], and may be reluctant to try eHealth service due to previous unsuccessful experiences [17,18]. Consequently, the online health care resource use rate among older adults is merely 15.2% [19]. Previous studies have demonstrated that an increased growth mindset is a potential key strategy for improving positive responses to eHealth because older adults with a growth mindset can improve age-related stereotypes, increase their willingness to adopt healthy behaviors and resilience in the face of difficulties, and tend to use eHealth more [20-23].

The growth mindset theory, proposed by Dweck and Leggett [24], theorizes that individuals possess one of two types of mindsets—fixed mindset and growth mindset—regarding the malleability of abilities, intelligence, and other attributes. Individuals with fixed mindsets view the attributes as unchangeable and uncontrollable internal parameters, and often withdraw or abandon efforts when faced with difficulties. Conversely, growth mindset individuals believe the attributes are malleable and controllable, and they always view failure as an opportunity to learn and improve [25]. In addition, the literature suggests that a mindset intervention is particularly effective in changing behavior by improving participants’ persistence in goal-oriented behavior [26]. Therefore, the eHealth mindset for an intervention program appeared to be a promising approach.

Many studies have shown that interventions fostering a growth mindset are beneficial to behavior change, such as improving performance in students [27-29]. mitigating weight gain resulting from dieting setbacks [30], controlling healthy eating and exercise in obese youth [31], and smoking cessation [23]. In the geriatric population, existing studies have shown that a growth mindset is significantly associated with intention to engage in health behavior [32], cognitive function [33], and successful aging [34]. Experimental studies have demonstrated its benefits in improving age-related stereotypes [35] and memory performance [36] as well. Dang and Liu [37] have provided evidence that individuals who believe human mental capacity is incremental report more positive feelings about robots and show higher willingness to interact with them.

In summary, previous studies have investigated the growth mindset in different populations and have demonstrated potential relationships between growth mindset and positive responses to eHealth. Studies have also been conducted on the growth mindset levels of older adults, the results have demonstrated that this aspect needs to be improved [20,38], and that growth mindset may be a key target in bridging the “digital divide” among older adults. However, most of these studies were cross-sectional and descriptive, making it difficult to provide direct guidance for practice. Therefore, this study was conducted to develop and investigate the effectiveness of a growth mindset intervention based on growth mindset theory. It aimed to improve the levels of growth mindset and knowledge, willingness, belief, and practice of smart medicine among older patients with chronic disease in the context of global trends in aging and chronic diseases.

## Methods

### Design

This study used a quasi-randomized controlled trial design conducted over 12 weeks. Before conducting the study, the researchers numbered the wards of internal medicine departments (excluding intensive care units) according to the order of bed number. The wards were then randomly divided into intervention wards and control wards using a random number generator. Patients were recruited to the control group (eHealth only) in the control ward and the intervention group (growth mindset intervention + eHealth) in the intervention unit. Participants in the control group could undergo the training after the study concluded. The study was registered on ClinicalTrials.gov (NCT06550817).

### Setting and Participants

Participants (N=77) were enrolled from a hospital internal medicine department in Hangzhou, Zhejiang Province in China between September 2021 and October 2022. The inclusion criteria were: (1) participants aged 60 years and older, (2) diagnosed with ≥1 chronic disease, (3) owned at least one smartphone, (4) ability to communicate clearly in writing or orally, and (5) willingness to participate in this study. The exclusion criteria were: (1) inability to use WeChat (Tencent Holdings Limited) or phone to communicate, (2) diagnosed as having a mental illness or cognitive impairment, (3) acute severe disease or loss of self-care ability, and (4) concurrent participation in other similar training.

This study used a quasi-experimental design, and the sample size was calculated based on comparing the means of the two samples [39]. An a priori power analysis showed that 34 participants per group (total N=68), accounting for anticipated attrition, would achieve 90% power at a 5% significance level to detect the expected mean difference. Finally, 77 participants started the intervention, and 74 completed the 12-week intervention period, with an attendance rate of 96.10% (74/77).

### Ethical Considerations

The ethics committee of the School of Nursing at Hangzhou Normal University has approved this study (2022015). Informed consent was obtained from all participants involved in this study. Participants were informed of the study’s purpose, methods, and process, as well as their right to withdraw at any time. Only the relevant data of the participants were used for this study and all collected data were pseudonymized to protect participants’ confidentiality. As compensation for study participation, participants received a ¥50 (US $6.95) supermarket voucher and a vegetable package upon study completion.

### Blinding and Randomization

The single-blind principle was strictly applied in this study. Neither the study participants nor the data collectors had access to the grouping and the content of the intervention, thus mitigating subjective bias. Patients were recruited into the control group in the control ward and into the intervention group in the intervention ward. Random grouping by ward avoided cross-contamination when patients in the same ward were divided into different groups. Participants were debriefed at the end of the study regarding the full purpose and differences between groups in the study.

### Intervention

The eHealth training program was delivered to participants by nursing students with a master’s degree, who have received training in growth mindset knowledge, and training related to the construction and implementation of nursing training studies. Psychological counselors, professors in geriatric nursing, and clinical experts in geriatric nursing served as consultants. The purpose, duration, and main contents of the training were introduced to the patients by the faculty before the training. Patients were asked about their learning experience and whether they were confused about the content of the lectures after the training. The faculty used the video training platform to track patients’ learning, and for those who did not complete the training in time, telephone communication was implemented to encourage them to improve their learning progress. This ensured that the patients mastered the content of the training and assistance was offered in solving problems.

#### Growth Mindset Intervention

Based on the previous literature review, this study found that previous intervention [27], deeply aligned with the growth mindset theory. The program is mature and verified by large samples in multiple regions, and hence, based on the framework of the intervention, the following 3 strategies were used for creating the intervention content: recognize growth mindset, explore growth mindset deeply, and apply growth mindset. The purpose of “recognize growth mindset” was to convince patients that they could still change their intelligence through learning, strengthening the belief that their brain can be exercised. The purpose of “explore growth mindset deeply” was to increase patients’ understanding of growth mindset and promote their willingness to cultivate a growth mindset. The purpose of “apply growth mindset” was for patients to learn to apply a growth mindset and connect it to real life. In addition, some studies have noted that there are adaptation issues in the implementation of the growth mindset [40]. The physical and mental functions of older adults show an irreversible decline trend, thus the cultivation and adaptation of the growth mindset in older adults are more difficult. Therefore, this study takes the characteristics of older adults into account and adds the theme “growth mindset in life” under the guidance of ife span theory by Baltes et al [41], to further cultivate the habit of using growth mindset in practice in older patients with chronic diseases. In this manner, the above 4 strategies were identified for the study.

Research shows that the introduction of brain science, story rewriting, skill training, and other methods can enhance the subjects’ identification and internalization of the growth mindset [42,43], so the research group formulated the intervention program based on this and the actual situation of older patients with chronic diseases. Table 1 shows the specific content. The intervention duration is 12 weeks. Themes 1‐6: once a week, online intervention is implemented and it lasts about 20 minutes each time, while face-to-face intervention lasts about 30 minutes each time, and they are carried out for a total of six weeks; theme 7: face-to-face or telephone intervention is implemented once every two weeks for about 15 minutes each time, and it is carried out for a total of six weeks.

**Table 1.** Implementation form of the growth mindset intervention program.

Intervention strategy	Intervention theme	Intervention content	Intervention form
Recognize growth mindset	Is intelligence innate?	Introduced the structure and function of the brain and neurons. Explained the plasticity of the brain. Emphasized the importance of learning and challenging new things. Strengthened the belief of “exercising the brain” and “the more the brain is used, the more flexible it is.”	Video lessons
	Can older adults become smart?	Introduce the changes in brain structure and function in older adults. List relevant scientific research to encourage patients to believe that the brain of older adults is still plastic. Encourage patients to talk about the relationship between “the plasticity of older adults’ brain” and their own intelligent medical.	Video lessons and face-to-face interaction
Explore growth mindset deeply	The 2 different mindsets	Introduce the concept of growth mindset. Share different mindsets in the process of learning eHealth, and guide patients to understand different behaviors, and results of the 2 mindsets. Emphasize the benefits of the growth mindset for older adults. Encourage patients to rewrite stories from the perspective of the growth mindset.	Video lessons, telelecture, and story rewriting
	Facing difficulties and challenges	Encourage patients to share their experiences of overcoming difficulties and guide them to discover the importance of a growth mindset. Make a case of the common difficulties and challenges of older adults with chronic diseases in the use of eHealth. Ask patients to imagine how to solve the problem through efforts, strategies, seeking help, and other ways if they are the parties.	Experience sharing and case studies
Apply growth mindset	The 4 steps of growth mindset	Introduce Dweck’s 4 step strategy to change the mindset: accept, observe, name, and educate. Encourage patients to describe the experience of the emergence of a fixed mindset during eHealth use and discuss how to implement the 4 steps of the growth mindset.	Video lessons and telelecture
	Growing mindset habit cultivation plan	Review the interventions that have been implemented. Encourage patients to talk about the challenges related to eHealth, and guide them to think about how to use a growth mindset to face the challenges. Complete the “growth mindset habit cue card.”	Face-to-face interaction
Growth mindset in life	Growth mindset in life	Encourage patients with good application of the growth mindset, and help patients with difficulties to solve problems.	Telephone follow-up

#### eHealth Program

The eHealth program intervention aimed to increase patients’ knowledge of eHealth and help them master common operational skills. The intervention forms included face-to-face instruction, video teaching, training manual, and telephone follow-up. Video teaching included “Smart Phone Basics, Online Registration, Mobile Phone Payment and Report Query,” “Introduction and Operation of Online Health Consultation,” “Introduction and Operation of Online Prescriptions and Residents’ Electronic Health Archives,” “Introduction and Operation of Hospital Online Disease Management,” “Methods to Obtain and Identify Reliable Health Knowledge,” and “Introduction to Wearable Mobile Medical Devices.” The training manual was a video supporting content, which was used with the videos. Finally, telephone follow-up was conducted to understand the learning situation and difficulties of the participants. The program lasted for 12 weeks. In the first 6 weeks, face-to-face eHealth teaching in the internal medicine department and telephone follow-up were conducted once a week, for about 10 minutes each time. In the last 6 weeks, only telephone follow-up was conducted.

### Measures

#### Implicit Theories of Intelligence Scale-6 (ITIS-6)

The Implicit Theories of Intelligence Scale-6 (ITIS-6) [44] was used to assess participants’ level of growth mindset. Participants completed the scale at baseline, 6, and 12 weeks of intervention. The scale contains 3 growth mindset items and 3 fixed mindset items. The Likert-6 scoring method was used, and a reverse scoring method was adopted for the fixed mindset dimension, with a higher score on the scale signifying a higher level of growth mindset. In this study, the Cronbach α coefficient for ITIS-6 was 0.95, indicating good internal consistency.

#### Questionnaire of Knowledge, Willingness, Confidence, and Practice of Smart Medicine (KWCP-SM)

According to the relevant policy documents on the promotion and implementation of eHealth, this study invited 5 experts with 10 years or more of work experience (including 2 clinical nurses, 1 community nurse, and 2 community doctors) to conduct face-to-face interviews, and developed the questionnaire of knowledge, willingness, confidence, and practice of smart medicine. The final questionnaire had the 4 dimensions of knowledge, willingness, confidence, practice, and 9 items including online registration, and mobile phone payment. There were 36 items in total. The questionnaire adopted the Likert-5 scoring method. The higher score indicated a higher level of knowledge, willingness, confidence, and practice of smart medicine. The Cronbach α coefficient for the scale was 0.97, indicating good internal consistency.

### Statistical Analysis

Baseline demographic and medical characteristics within groups were compared with an independent-sample *t* test (for quantitative variables), *χ*2 test (qualitative variables), or nonparametric rank-sum test (qualitative variables following grade data). The Mann-Whitney *U* test was applied to assess the difference in growth mindset and KWCP-SM between the two groups because of their abnormal distributions. Within-group comparisons were performed by the Wilcoxon signed ranks test. To evaluate the between-group differences over time, a generalized estimation equation (GEE) was performed. Statistical analysis was carried out with SPSS 25.0 version (IBM). *P*<.05 was considered statistically significant.

## Results

### Participants

**Figure 1.**
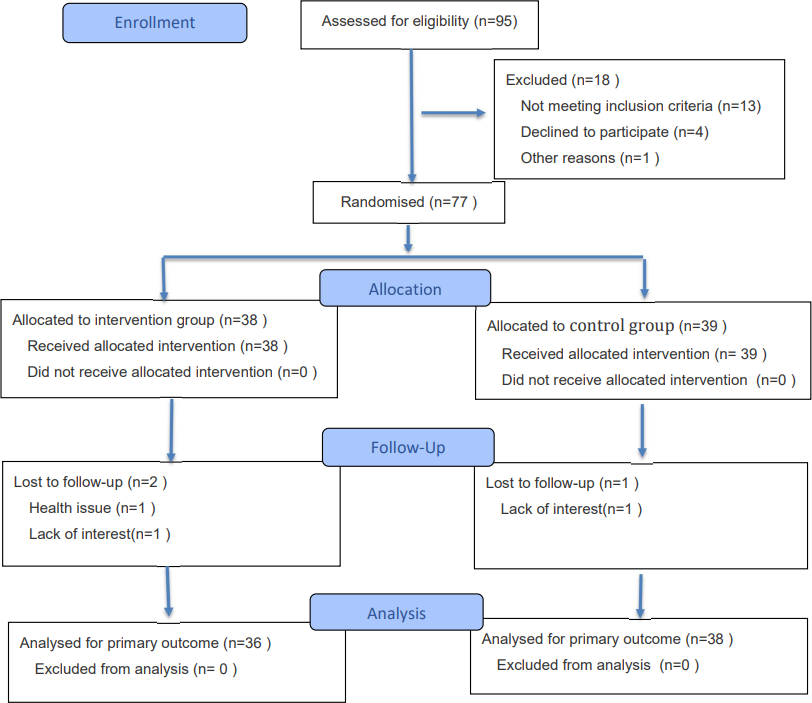
CONSORT (Consolidated Standards of Reporting Trials) flow chart of participation in the eHealth training program.

Out of the 77 participants who participated in the intervention, 74 completed the 12-week intervention period, resulting in an attendance rate of 96.10% (74/77), with 36 in the intervention group and 38 in the control group. Among the 3 participants in the two groups who withdrew from the study, 2 participants in the intervention group reported health issues and lost interest, respectively, and 1 participant in the control group lost interest. Figure 1 shows the CONSORT (Consolidated Standards of Reporting Trials) diagram of the participant flow and the EHEALTH checklist is provided (see Checklist 1). Table 2 shows the baseline characteristics of participants. No significant differences were observed between the intervention and control groups regarding demographic and medical characteristics, growth mindset, and KWCP-SM of participants.

**Table 2.** Baseline demographics, growth mindset, and knowledge, willingness, confidence, and practice of smart medicine (KWCP-SM) of participants.

Characteristic	Intervention (n=36)	Control (n=38)	Z, *t* test, or χ^2^(df)	*P* value
Demographics				
Age (years), mean (SD)	67.47 (7.44)	67.03 (6.64)	0.27^a^(72)	.53
Sex, n (%)			2.60^b^(1)	.16
Male	18 (50)	26 (68.42)		
Female	18 (50)	12 (31.58)		
Religious belief			2.81^b^(1)	.11
Have	12 (33.33)	20 (52.63)		
Do not have	24 (66.67)	18 (47.37)		
Educational level			−0.63^c^	.53
Primary school and below	15 (41.67)	14 (36.84)		
Middle school	14 (38.89)	14 (36.84)		
High school	5 (13.89)	7 (18.42)		
Junior college and above	2 (5.56)	3 (7.89)		
Marital status			0.35^b^(1)	.74
Married	32 (88.89)	32 (84.21)		
Others (including divorced, etc.)	4 (11.11)	6 (15.79)		
Place of residence			0.45^b^(1)	.64
Urban	17 (47.22)	15 (39.47)		
Rural	19 (52.78)	23 (60.53)		
Occupation (previous main occupation if retired)			3.10^b^(5)	.71
Public institution personnel	4 (11.11)	6 (15.79)		
Enterprise employee	10 (27.78)	13 (34.21)		
Worker	9 (25)	5 (13.16)		
Farmer	9 (25)	9 (23.68)		
Self-employed	3 (8.33)	2 (5.26)		
Others (including freelance, etc.)	1 (2.78)	3 (7.89)		
Monthly income (RMB)			−0.60^c^	.55
<1000（<US $139.10)	5 (13.89)	4 (10.53)		
1000‐2999（US $139.10‐US $417.14)	7 (19.44)	7 (18.42)		
3000‐4999（US $417.28‐US $695.33)	13 (36.11)	13 (34.21)		
>5000（>US $695.47)	11 (30.56)	14 (36.84)		
Living places			2.94^b^(4)	.57
Living with a spouse and children	13 (36.11)	11 (28.95)		
Living with a spouse	20 (55.56)	19 (50)		
Living with children	1 (2.78)	4 (10.53)		
Living in institutions (including nursing home, etc)	1 (2.78)	1 (2.63)		
Living alone	1 (2.78)	3 (7.89)		
Health insurance			2.05^b^(3)	.56
Urban resident basic medical insurance	17(47.22)	22(57.89)		
New rural cooperative medical scheme	16(44.44)	11(28.95)		
Commercial medical insurance	1(2.78)	2(5.26)		
No medical insurance	2(5.56)	3(7.89)		
The number of comorbidities			−0.31^c^	.76
1	12(33.33)	9(23.68)		
2	13(36.11)	19(50)		
≥3	11(30.56)	10(26.32)		
Self-rated health status (0‐10), mean (SD)	6.32 (1.72)	5.91 (2.05)	−0.54^a^(72)	.60
ITIS^d^ score, mean (SD)	18.50 (8.65)	17.53 (9.09)	−0.47^a^(72)	.81
KWCP-SM^e^ score, mean (SD)	80.06 (22.25)	79.58 (28.05)	−0.32^f^	.75
Knowledge score, mean (SD)	23.75 (7.24)	23.47 (8.21)	−0.52^f^	.60
Willingness score, mean (SD)	23.19(7.63)	23.29 (9.46)	−0.06^f^	.95
Confidence score, mean (SD)	20.56 (8.47)	20.74 (9.68)	−0.19^f^	.85
Practice score, mean (SD)	12.56 (4.77)	12.08 (4.72)	−0.23^f^	.82

a Denotes the independent-samples *t* test (t).

b Denotes the *χ*² test (*χ*²).

c Denotes the Wilcoxon or Mann-Whitney Z test (Z).

d ITIS: implicit theories of intelligence scale.

e KWCP-SM: knowledge, willingness, confidence, and practice of smart medicine.

f Denotes the Mann-Whitney *U* test (Z).

### Growth Mindset

In terms of growth mindset level, there was no significant difference between the two groups at baseline (*P*>.05), but there was a significant difference between the two groups after intervention (*P*<.05). The intervention group demonstrated a significant improvement in group changes in growth mindset at 12 weeks (9.89% change from baseline; *z* score=−5.13, *P*<.05), while no statistically significant change was observed in the control group (2.68% change from baseline; *z* score=−5.13, *P*>.05). The GEE analysis revealed the growth mindset changes over time in the intervention and the control groups. A significant group × time interaction was observed at 12 weeks of intervention (Wald=11.57; *P*<.05), indicating that there was a significant improvement in the intervention group than the control group.

### KWCP-SM

Table 3 shows the mean, SE, percentage change of outcomes, and the results of the Wilcoxon signed ranks test. Table 4 shows the GEE results of outcome valuables over time between the two groups. Regarding knowledge of smart medicine, at 12 weeks both the intervention group (29.35% change from baseline; *z*=−5.20; *P*<.001) and control group (27.06% change from baseline; *z*=−4.85; *P*<.001) had statistically significant within-group changes, but the difference between groups (Wald=1.14; *P*=.57) was not significant.

For willingness to use smart medicine, at 12 weeks the intervention group (19.53% change from baseline; *z* score=−3.25, *P*=.001) showed a marked improvement compared with baseline score, while the control group (1.12% change from baseline; *z* score=−0.67; *P*=.50) showed a slight decrease. The GEE analysis revealed a significant interactive effect between time and groups (Wald=10.63; *P*=.005).

For confidence in smart medicine, both groups experienced improvement during the 12-week intervention, while this improvement was larger in the intervention group (30.25% change from baseline; *z* score=−3.75; *P*<.001) than the control group (5.45% change from baseline; *z* score=−1.77; *P*=.08). In addition, a significant difference in group × time interactive effect was observed (Wald=25.09; *P*<.001).

For the practice of smart medicine, both the intervention group (6.61% change from baseline; *z* score=−1.69; *P*=.09) and control group (4.14% change from baseline; *z* score=−1.50; *P*=.13) showed a slight improvement at 12 weeks compared with baseline. However, no significant between-group difference was observed (Wald=1.51; *P*=.47).

**Table 3.** Comparison of pre- and postintervention mean of outcomes (growth mindset; knowledge, willingness, confidence, and practice of smart medicine [KWCP-SM]).

Variables	Groups	T0^a^, mean (SD)	T1^a^, mean (SD)	T2^a^, mean (SD)	Relative to baseline, %	*z* score	*P* value^b^
Growth mindset, ITIS^c^	IG^d^	18.50 (8.65)	21.89 (7.99)	20.33 (8.53)	9.89	−5.13	<.001
	CG^e^	17.53 (9.09)	17.79 (10.03)	18.00 (9.16)	2.68	−5.13	>.001
Knowledge, KWCP-SM^f^	IG	23.75 (7.24)	31.67 (5.13)	30.72 (6.09)	29.35	−5.20	<.001
	CG	23.47 (8.21)	31.61 (6.82)	29.82 (7.45)	27.06	−4.85	<.001
Willingness, KWCP-SM	IG	23.19 (7.63)	29.69 (7.52)	27.72 (8.16)	19.53	−3.25	.001
	CG	23.29 (9.46)	24.92 (8.55)	23.55 (7.13)	1.12	−0.67	.50
Confidence, KWCP-SM	IG	20.56 (8.47)	28.64 (8.23)	26.78 (9.13)	30.25	−3.75	<.001
	CG	20.74 (9.68)	21.87 (9.45)	21.87 (9.45)	5.45	−1.77	.08
Practice, KWCP-SM	IG	12.56 (4.77)	14.56 (5.57)	13.39 (5.18)	6.61	−1.69	.09
	CG	12.08 (4.72)	13.21 (5.31)	12.58 (4.79)	4.14	−1.50	.13

a Time points. T0: baseline; T1: 6-week; T2: 12-week.

b Within-group changes were examined with Wilcoxon signed ranks test.

c ITIS: Implicit theories of intelligence scale.

d IG: intervention group.

e CG: control group.

f KWCP-SM: Questionnaire of knowledge, willingness, confidence, and practice of smart medicine.

**Table 4.** Effects of intervention on outcome variables between two groups: Growth mindset and knowledge, willingness, confidence, and practice of smart medicine (KWCP-SM).

Outcomes	Group		Time		(Group × time)^a^	
	Wald	*P* value	Wald	*P* value	Wald	*P* value
Growth mindset	1.63	.20	15.84	<.001	11.57	.003
Knowledge	0.08	.78	204.28	<.001	1.14	.57
Willingness	3.26	.07	26.53	<.001	10.63	.005
Confidence	3.89	.049	40.95	<.001	25.09	<.001
Practice	0.68	.41	19.47	<.001	1.51	.47

a Results of growth mindset and knowledge, willingness, confidence, and practice of smart medicine (KWCP-SM) outcome analysis using the generalized estimation equation (GEE).

## Discussion

### Principal Findings

This study constructed a growth mindset intervention program for older patients with chronic diseases and evaluated its effects on their growth mindset and positive response to eHealth. After 12 weeks of intervention, the findings showed a significant improvement in the total ITIS-6 and KWCP-SM scores of patients in the intervention group compared with the control group, which suggests that the intervention was effective. We tailored our growth mindset program to address the mindset and micro-psychology of older adult patients with chronic diseases. This approach offers a new direction in bridging the digital divide targeting older adults.

### Comparison With Previous Work

While the intervention program of this study effectively improves the growth mindset of older adults with chronic diseases,research on the effectiveness of growth mindset interventions in increasing the level of growth mindset in different populations is not entirely consistent. For example, Plaks and Chasteen [36] conducted a growth mindset psychological experiment with 88 older adults and found significant increases in the growth mindset in the experimental group. Orvidas et al [31] conducted an online growth mindset intervention with 48 obese children and adolescents and showed that the experimental group had better levels of growth mindset than the control group and significantly improved perceptions of health behaviors and self-efficacy. However, Sridharan et al [23] conducted an online growth mindset intervention with 398 smokers for 24 days and found that there was no significant difference in the level of growth mindset between the intervention group and the control group after the intervention, which may be due to the excessive amount of growth mindset intervention content provided to the participants. In this study, the intervention group showed better improvement in growth mindset than the control group, which could be attributed to the following reasons: First, we consulted previous growth mindset interventions that worked and adopted the framework of the intervention program previously published [27] as the main theme. At the same time, we combined the characteristics of psychological dependence of older adults to construct the themes, a total of 4 strategies along with the themes were included. The “Recognizing growth mindset” strategy used video lessons and face-to-face interaction to let the participants believe the plasticity of the brain of their own; the “Exploring growth mindset deeply” strategy used video lessons, telelecture, and story rewriting to promote deeply understanding and internalization of the growth mindset; the “Applying growth mindset” strategy used daily practice to promote the integration of growth mindset into the patient’s real life and learn to apply it; the “growth mindset in life” strategy used phone follow-up to help older adults with difficulties to solve problem and promote habit formation. Second, considering that effective training and dependence behavior are important measures for individual growth in old age [45], we helped patients identify the timing of a fixed mindset and adjust and cultivate a growth mindset through four steps: acceptance, observation, naming, and education. Furthermore, by teaching older adults about brain plasticity and the experience of growth mindset activities, this study, to a certain extent, broke the stereotype of “old age is frailty,” and instilled the hope of improvement in older adults. In addition, patients overcame difficulties through the growth mindset intervention and successfully mastered the experience of eHealth treatment, enabling them to realize that the growth of older adults is controllable, achievable, and recognized by society. This hopeful and emotional experience enhanced their belief that their ability can still be developed, improving their level of growth mindset.

After the 12-week eHealth program intervention, the total score of KWCP-SM, which measured the level of willingness and confidence in smart medicine, improved significantly in both the intervention and control groups compared to the pre-intervention period. This suggests that the traditional teaching model can improve the ability of older adults to use eHealth to some extent, and the result is consistent with the study by Zhao et al [46]. There are some possible explanations for this. First, existing studies have found that older adults experience major negative psychological emotions such as age-related stereotype, frustrated self-esteem, and dwindling motivation, which hinder the integration of eHealth in their lives [47,48]. Growth mindset intervention can effectively improve age-related stereotypes by instilling the belief in the plasticity of older adults’ intelligence and ability [49,50]. It also reduces patients’ excessive attention to learning outcomes, regards failure as a process of learning and improvement, protects their self-esteem and secondary fear of science and technology in the event of failure to a certain extent, and stimulates their internal motivation [51-53]. Second, the theory of growth mindset emphasizes that efforts, strategies, and timely help-seeking are important paths for learning and growth [27]. This study not only teaches patients to learn knowledge and skills, emphasizing that the key premise is hard work, but also empowers patients with eHealth learning strategies and help-seeking methods. Furthermore, our intervention met the practical needs of older adults who urgently need eHealth health care but are unable to take full advantage of it because they have not kept pace with technological developments. As a result, the willingness and interest of older people to learn are relatively high and the outcomes of this eHealth program are more positive.

### Strengths and Limitations

The growth mindset intervention in this trial was developed in a theoretical and evidence-based manner and contains a tailored education program taking the characteristics of the older adults with chronic diseases into account. In addition, the training program organically integrates local slang into the training, increases the patients’ trust in the plasticity of older adults’ brains and their belief in lifelong learning, and sets up regular and multiple nurse-patient communication sessions, which facilitate real-time interaction and feedback, thus meeting the patients’ personalized needs.

Several limitations of this study should be noted here. First, the study was carried out at only one comprehensive hospital in Hangzhou. More multicenter and large sample studies should be carried out to further verify the scheme’s effectiveness. Second, the between-group difference in actual eHealth behavior change was not statistically significant. This may due to time constraints, only the effect of the growth mindset intervention for 12 weeks was confirmed. In the future, research should continue to track participants for 12 weeks or more to identify the effectiveness of the intervention program over the long term. Third, this study took the lead in building a growth mindset intervention program for older patients with chronic diseases and preliminarily verified the effectiveness. However, during the intervention, individual differences were found among older adults which may partly explain the nonsignificant finding, so future studies need to constantly adjust and improve the intervention plan according to the characteristics and needs of the participants.

Future studies should also implement the double-blind method and increase the objective evaluation indicators related to eHealth (such as frequency of visiting doctors, compliance, clinical nursing outcomes, etc.) to verify the efficacy of this program. Finally, future studies can build a platform for elderly growth mindset intervention with emerging technologies to achieve large-scale and accurate intervention, to improve the level of growth mindset in older adults to a greater extent.

### Conclusions

The growth mindset intervention program for older adults with chronic diseases constructed in this study effectively improves the growth mindset of older adults with chronic diseases, enhances their mastery of eHealth knowledge, and boosts their confidence and willingness to use eHealth tools. It is expected to help patients meet their own diagnostic, treatment, disease control, and other practical needs through eHealth solutions. A growth mindset shows high potential in promoting eHealth use among older adults. This study is referable due to its creation of training content based on the growth mindset theory and investigation of the effect of the training on a range of variables. Further research is needed to improve the growth mindset intervention, in particular by considering older patients’ needs and interests, improving its adoption, and developing fault-tolerant and lifelong growth environments that enhance growth mindset among older adults with chronic diseases. The growth mindset intervention program in this study could also be applied to enhance growth mindset and eHealth behaviors among older adults with chronic diseases in other countries by integrating localized case examples, community specific customs, and beliefs.

## Supplementary material

10.2196/65519 Checklist 1 CONSORT-EHEALTH checklist V 1.6.1.

## Abbreviations

GEE: generalized estimation equation
ITIS-6: implicit theories of intelligence scale-6
KWCP-SM: knowledge, willingness, confidence, and practice of smart medicine
